# Mixed Ionic-Electronic Conductors Based on PEDOT:PolyDADMA and Organic Ionic Plastic Crystals

**DOI:** 10.3390/polym12091981

**Published:** 2020-08-31

**Authors:** Rafael Del Olmo, Nerea Casado, Jorge L. Olmedo-Martínez, Xiaoen Wang, Maria Forsyth

**Affiliations:** 1 Joxe Mari Korta Center, POLYMAT University of the Basque Country UPV/EHU, Avda. Tolosa 72, 20018 Donostia-San Sebastian, Spain; rafael.delolmo@polymat.eu (R.D.O.); jolmedo001@ikasle.ehu.es (J.L.O.-M.); 2 Institute for Frontier Materials (IFM), Deakin University, Geelong, VIC 3217, Australia; xiaoen.wang@deakin.edu.au; 3 IKERBASQUE, Basque Foundation for Science, 48011 Bilbao, Spain; 4 ARC Centre of Excellence for Electromaterials Science (ACES), Deakin University, Burwood, VIC 3125, Australia

**Keywords:** mixed ionic electronic conductors, polyelectrolytes, organic ionic plastic crystals, conducting polymers

## Abstract

Mixed ionic-electronic conductors, such as poly(3,4-ethylenedioxythiophene): poly(styrenesulfonate) (PEDOT:PSS) are postulated to be the next generation materials in energy storage and electronic devices. Although many studies have aimed to enhance the electronic conductivity and mechanical properties of these materials, there has been little focus on ionic conductivity. In this work, blends based on PEDOT stabilized by the polyelectrolyte poly(diallyldimethylammonium) (PolyDADMA X) are reported, where the X anion is either chloride (Cl), bis(fluorosulfonyl)imide (FSI), bis(trifluoromethylsulfonyl)imide (TFSI), triflate (CF_3_SO_3_) or tosylate (Tos). Electronic conductivity values of 0.6 S cm^−1^ were achieved in films of PEDOT:PolyDADMA FSI (without any post-treatment), with an ionic conductivity of 5 × 10^−6^ S cm^−1^ at 70 °C. Organic ionic plastic crystals (OIPCs) based on the cation N-ethyl-N-methylpyrrolidinium (C_2_mpyr^+^) with similar anions were added to synergistically enhance both electronic and ionic conductivities. PEDOT:PolyDADMA X / [C_2_mpyr][X] composites (80/20 wt%) resulted in higher ionic conductivity values (e.g., 2 × 10^−5^ S cm^−1^ at 70 °C for PEDOT:PolyDADMA FSI/[C_2_mpyr][FSI]) and improved electrochemical performance versus the neat PEDOT:PolyDADMA X with no OIPC. Herein, new materials are presented and discussed including new PEDOT:PolyDADMA and organic ionic plastic crystal blends highlighting their promising properties for energy storage applications.

## 1. Introduction

Inorganic mixed ionic-electronic conductors (MIECs) have been well studied and applied in different fields [1,2], as they show excellent ionic conductivities and thermal stability. However, in the last few decades, the potential of organic MIECs (OMIECs) has been highlighted because of their high electronic conductivity and flexibility in their synthesis and processability. Previous studies of inorganic MIECs illustrate the difficulties associated with OMIECs, with many factors affecting the ionic-electronic transport, such as the presence of solvent, mobile ions and temperature [3]. However, a growing number of OMIECs have been successfully applied in the field of energy storage systems (ESSs) [4], ion pumps [5], sensors [6], bioelectronics [7] and thermoelectrics [8].

OMIECs are predominated by π-conjugated polymers but also radical polymers and conjugated small molecule systems. Some of the π-conjugated polymers successfully employed are based on polyaniline, polypyrrole and poly(3,4-ethylenedioxythiophene) (PEDOT). Many studies have aimed to enhance the properties such as the electronic conductivity, by adding high boiling point solvents [9], superacids [10] or ionic liquids [11], mechanical properties [12] or employing hybrid architectures [13], even showing metallic conductivities of 6259 ± 1468 S cm^−1^ for thin post-treated films [14] and totally flexible foils [12]. These π-conjugated polymers can be either functionalized with polyelectrolytes and polymer electrolytes via pendant groups [15], or form copolymers and blends with other polymers (e.g., polyethylene oxide (PEO) [4] and polystyrene sulfonate (PSS) [12]), which provide the ionic conductivity. Nevertheless, very few studies have been carried out to enhance their weaker feature, that is the ionic conductivity in their dry state.

Among all of the conducting polymers, PEDOT is the most widely studied, due to its high electronic conductivity and thermal stability. PEDOT has been widely studied with a tosylate (PEDOT:OTs) [16] or chloride (PEDOT:Cl) anion [17], but polystyrene sulfonate (PSS) is the most commonly used anion. PSS acts as a dopant to promote the polaron and bipolaron charges within PEDOT, and acts as a dispersing agent to obtain a processable water dispersion. For example, the commercially available PEDOT:PSS dispersion, Clevios PH1000, has an electronic conductivity around 0.2 S cm^−1^. Moreover, there are other polymers that have shown excellent performance as ionic conductors like polyethers [4,18] and poly(diallyldimethylammonium) (PolyDADMA X) [19], but they have been rarely studied in combination with PEDOT to enhance the ionic conductivity of MIECs.

The figures of merit of the different applications are governed basically by ionic transport, electronic transport and ionic-electronic coupling. Different orders of conductivities are required to have acceptable performances in different aspects. For instance, in the field of ESSs either the specific energy (mWh g^−1^) or specific capacitance (F g^−1^) is controlled by the ionic-electronic coupling, whereas the specific power or the efficiency at high charging rates are mostly dominated by ionic transport, which is typically slower than electronic transport. In transistors and sensors, the sensitivity depends equally on the ionic and electronic transport and hence the ionic-electronic coupling; on the other hand the ionic transport limits the response time (s) [1]. To obtain devices with a better actuation, electronic conductivities around 10^1^–10^2^ S cm^−1^ and ionic transports of 10^−4^ S cm^−1^, close to solid electrolytes, should be the goal for those mixed conductors. Different strategies can be taken to achieve these values, namely either the modification of the conducting polymer or making adjustments with different stabilizers or additivies.

The aim of this work was to develop a new series of OMIECs based on PEDOT and poly(diallyldimethylammonium) polymers with different anions. The anion plays a key role in both the electronic conductivity of PEDOT [20] and in the ionic conductivity of poly(diallyldimethylammonium). Therefore, in the first part of this work, a series of poly(diallyldimethylammonium X) (PolyDADMA X) materials were prepared, where X is either chloride (Cl), bis(fluorosulfonyl)imide (FSI), bis(trifluoromethanesulfonyl)imide (TFSI), trifluoromethanesulfonate (CF_3_SO_3_) or p-toluenesulfonate (Tos). The PolyDADMA X materials were then combined with PEDOT to form OMIECs, to observe the effect of the anion on the ionic and electronic conductivity, thermal behavior and electrochemical performance.

In the second part of this work, composites of PEDOT:PolyDADMA X with organic solid salts based on the N-ethyl-N-methylpyrrolidnium (C_2_mpyr^+^) cation with the analogous X anions (FSI, TFSI, CF_3_SO_3_ and Tos) were prepared. This was motivated by previous studies based on the use of ionic liquids (ILs) as electronic dopants of PEDOT, which reported high electronic conductivities [21,22]. While [C_2_mpyr][FSI] and [C_2_mpyr][TFSI] have been widely studied and applied in ESSs [23,24], herein the synthesis and characterization of [C_2_mpyr][CF_3_SO_3_] and [C_2_mpyr][Tos] is also presented. These salts, or organic ionic plastic crystals (OIPCs), are considered the solid-state cousins of ILs. The materials can possess plastic behavior and high ionic conductivities. In previous works, Nti et al. [25] recently reported their study on the interactions between OIPCs and polymers, such as poly(vinylidene fluoride) (PVDF) and polystyrene (PS), which showed that the combination of OIPCs and polymer particles enhanced the ionic conductivity and resulted in good mechanical stability of the composites. Therefore, in this article the potential of organic ionic plastic crystals to enhance both the ionic conductivity and the mechanical properties of the PEDOT:PolyDADMA OMIECs was investigated.

## 2. Materials and Methods

All the characterization methods and their specifications are in SI.

### 2.1. Materials

Poly(diallyldimethylammonium chloride) (PolyDADMAC) (*M*_w_ = 400,000–500,000 g mol^−1^) 20 wt% in water, p-toluenesulfonic acid monohydrate (Tosylic acid) (98.5%) and lithium(I) bis(trifluoromethanesulfonyl)imide (LiTFSI) (99.9%) were purchased from Sigma-Aldrich. Potassium bis(fluorosulfonyl)imide (KFSI) (98%) and N-ethyl-N-methylpyrrolidinium bromide (C_2_mpyrBr) (99%) were supplied by abcr GmbH. Trifluoromethanesulfonic acid (TFMSA) (98%) was supplied by Alfa Aesar. 3,4-ethylenedioxythiophene (EDOT) (99%) and Amberlite™ IRN-78 ion-exchange resin, OH-form were purchased from Fisher Scientific. 1-ethyl-1-methylpyrrolidinium bis(trifluoromethylsulfonyl)imide, 99% was supplied by IoLiTec. Dialysis tubing of regenerated cellulose, pre-treated, 38 mm MWCO 1 kD (1000 Daltons) and hydrochloric acid (HCl) (37%) were purchased from Scharlab. 

### 2.2. Synthesis of [C_2_mpyr][FSI]

The synthesis of [C_2_mpyr][FSI] was carried out following a previously reported, similar procedure [26]. An amount of 9.75 g (0.050 mol) of [C_2_mpyr][Br] and 12.53 g (0.057 mol) of KFSI were separately dissolved, each in 40 mL of distilled water. Then, the KFSI solution was added dropwise into the [C_2_mpyr][Br] solution with vigorous stirring. [C_2_mpyr][FSI] was obtained as a white precipitate that was purified after a liquid-liquid separation with dichloromethane (DCM). After drying under vacuum overnight at 60 °C a waxy solid of [C_2_mpyr][FSI] was obtained.

### 2.3. Synthesis of PolyDADMA FSI and PolyDADMA TFSI

PolyDADMA FSI and PolyDADMA TFSI were synthesized by the anionic exchange reaction of PolyDADMAC in the presence of the fluoronated salt (LiFSI and KTFSI), to synthesize the required polymer as previously reported [27]. In both cases, after filtration of the precipitate, a white solid powder was obtained.

### 2.4. Synthesis of [C_2_mpyr][CF_3_SO_3_], [C_2_mpyr][Tos], PolyDADMACF_3_SO_3_ and PolyDADMA Tos

These organic salts and polymers were synthesized via a two-step anion exchange reaction, following a previously reported, similar procedure [28]. Briefly, an aqueous solution of [C_2_mpyr][OH] was prepared by passing an aqueous solution of [C_2_mpyr][Br], which has a similar viscosity as water, through a column filled with anion exchange resin (SUPELCO AMBERLITE IRN-78) in the hydroxide form. After that, [C_2_mpyr][OH] was neutralized by the dropwise addition of the equimolar acid aqueous solution (TFMSA and Tosylic acid) to obtain the required compound, using an ice bath for cooling. The obtained solutions were stirred at ambient temperature and pressure for 12 h. Excess water was then removed by rotatory evaporation under vacuum. The same procedure was used for PolyDADMA CF_3_SO_3_ and PolyDADMA Tos, using the acids TFMSA and tosylic acid. The chemical structures of the prepared polyelectrolytes were confirmed by ^1^H and ^9^F NMR analysis (see Figures S2–S7).

### 2.5. Synthesis of PEDOT:PolyDADMA FSI and PEDOT:PolyDADMA TFSI

The synthesis of PEDOT:PolyDADMA FSI and PEDOT:PolyDADMA TFSI were carried out following a previously reported procedure [29], by oxidative polymerization in an acidic medium. It was previously reported that using an acidic medium can enhance the doping of PEDOT chains [30]. EDOT (0.3 mL, 2.81 mmol) and 20 wt% PolyDADMAC (1.92 mL, 2.48 mmol) were dispersed in 50 mL of 0.1 M HCl aqueous solution. In a second flask, ammonium persulfate (APS) (0.96 g, 4.22 mmol) was dissolved in 20 mL of 0.1 M HCl. After 20 min of bubbling in an N_2_ atmosphere to remove oxygen traces, the APS solution was added dropwise over a period of 5 min in an inert atmosphere and the color of the solution changed from clear to white and then to light blue as the reaction was occurring. After 24 h of reaction using an ice bath, the typical dark blue color of PEDOT was observed in the solution.

Subsequently, the fluorinated salt (LiFSI or KTFSI) was added in excess in order to precipitate PolyDADMA as PolyDADMA FSI or PolyDADMA TFSI and dope the PEDOT. PEDOT:PolyDADMA FSI and PEDOT:PolyDADMA TFSI were obtained after filtration and washing with Milli-Q water. In both cases the molar ratio of PEDOT:PolyDADMAX was 1.14:1.

### 2.6. Synthesis of PEDOT:PolyDADMA Cl, CF_3_SO_3_ and PEDOT:PolyDADMA Tos

The synthesis of PEDOT:PolyDADMA Tos was performed by polymerization of the EDOT monomer (0.33 mL, 3.05 mmol) in 30 mL of 0.1 M tosylic acid in the presence of the previously synthesized PolyDADMA Tos (0.8 g, 2.68 mmol). After adding APS (1.04 g, 4.58 mmol) as the oxidant and after 24 h of reaction under an inert atmosphere as previously explained, a dark blue dispersion was observed. The APS and excess acid were removed over 2 days of dialysis in regenerated cellulose membranes of 1000 Da. After 2 days of freeze drying, the desired compound was obtained as a dark blue sponge-like solid. The same procedure was followed to obtain PEDOT:PolyDADMA Cl (using hydrochloric acid and the commercially available PolyDADMAC) and PEDOT:PolyDADMA CF_3_SO_3_ (using TFMSA as the acid, and using the previously synthesized PolyDADMA CF_3_SO_3_). In the case of the CF_3_SO_3_ system, it was necessary to first dissolve the PolyDADMA CF_3_SO_3_ in the minimum quantity of dimethyl sulfoxide (DMSO) prior to adding the acid and EDOT monomer. In all cases the molar ratio of PEDOT:PolyDADMA X was 1.14:1.

## 3. Results and Discussion

**Figure 1:**
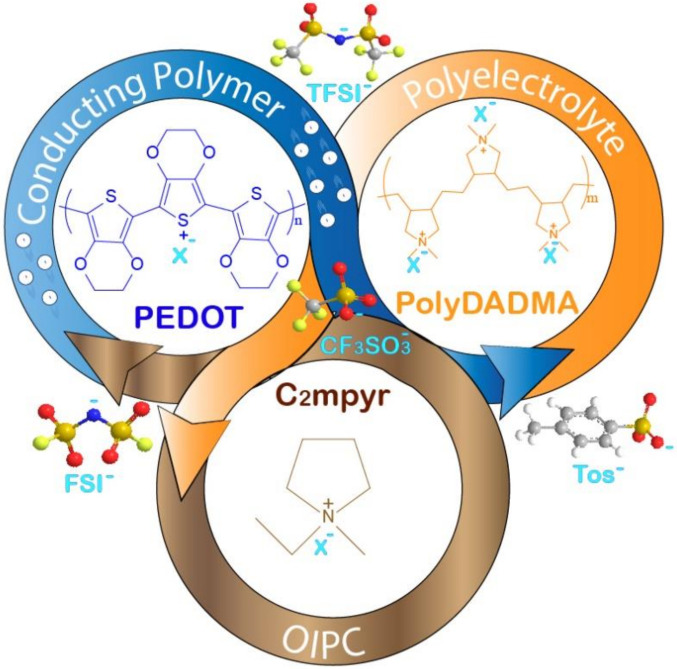
Scheme of the ternary system studied in this work.

In this article we investigated the ternary system between PEDOT, PolyDADMA and pyrrolidonium OIPC, as illustrated in Figure 1, in the presence of four different counter-anions, including triflate, FSI, TFSI and Tosylate. For this purpose, first we reported the synthesis and characterization of the individual systems, in the form of neat PolyDADMA X. Secondly, the synthesis and characterization of PEDOT:PolyDADMA was reported. Thirdly, the characterization of the OIPC was reported and finally the ternary system was investigated.

### 3.1. Neat PolyDADMA X

**Figure 2:**
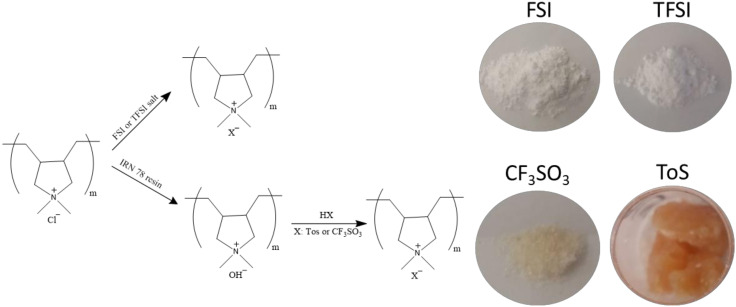
Synthesis of different polyelectrolyte poly(diallyldimethylammonium) (PolyDADMA X) materials.

A series of PolyDADMA X materials were synthesized via anion exchange of PolyDADMAC, where X is either FSI, TFSI, CF_3_SO_3_ or Tos, as explained above, following the scheme of the Figure 2. The thermal behavior and ionic conductivity of these compounds were tested and are discussed below.

#### 3.1.1. Thermal Analysis

Thermogravimetric analysis (TGA) of the PolyDADMA samples was conducted to investigate their thermal stabilities. This technique can result in an overestimate of the long-term thermal stability compared to an isothermal measurement [31], but it is nevertheless a valuable initial assessment of the relative impact of the different anions on the thermal properties. 

**Figure 3:**
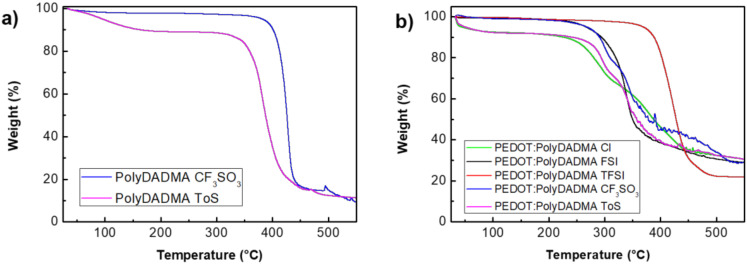
(**a**) Thermogravimetric analysis (TGA) curves of the neat PolyDADMA CF_3_SO_3_ and Tos polyelectrolytes and (**b**) TGA curves of PEDOT:PolyDADMA Cl, FSI, TFSI, CF_3_SO_3_ and Tos polyelectrolytes. PolyDADMA = poly(diallyldimethylammonium); CF_3_SO_3_ = triflate; Tos = tosylate; PEDOT = poly(3,4-ethylenedioxythiophene); Cl = chloride; FSI = bis(fluorosulfonyl)imide; TFSI = bis(trifluoromethylsulfonyl)imide.

PolyDADMA FSI and PolyDADMA TFSI have been previously studied [31]; they showed a decomposition temperature (*T*_d_) of 300 °C and 450 °C, respectively. In this study, the thermal behavior of PolyDADMA CF_3_SO_3_ and PolyDADMA Tos was studied by TGA, as shown in Figure 3a. The TGA curves show that the degradation of PolyDADMA CF_3_SO_3_ and PolyDADMA Tos occur in one step at temperatures higher than 427 °C and 382 °C, respectively. Therefore, the decomposition temperature of the different polyDADMA-X polyelectrolytes follows the trend: FSI < Tos < CF_3_SO_3_ < TFSI, which shows a huge impact of the anion on the thermal stability as previously observed in ionic liquids with the same tendency [32]. It should be mentioned that a 10% weight loss related to absorbed water was observed for PolyDADMA Tos at around 100 °C, due to its hygroscopic nature.

As no transition temperatures were observed by differential scanning calorimetry (DSC) in the range of –70 and 200 °C, dynamic mechanical analysis (DMA) was used to study the thermal transitions of PolyDADMA X polyelectrolytes between 40 and 200 °C [31] (Figure S1). During DMA measurements, with increasing of temperature, a sharp decay in the storage modulus is usually observed when the material passes through the glass transition, thus *T*_g_ can be determined from the maximum of tan delta and the concomitant decrease in storage modulus [33]. In this work, from these DMA data, the *T*_g_ measured for PolyDADMA CF_3_SO_3_ is 154 °C and 152 °C for PolyDADMA Tos. The *T*_g_ of PolyDADMA FSI and PolyDADMA TFSI have been previously reported at 121 and 116 °C, respectively [31]. 

#### 3.1.2. Ionic Conductivity

**Figure 4:**
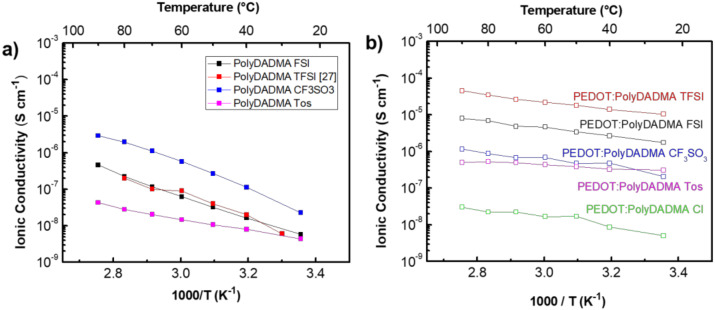
(**a**) Ionic conductivity of neat PolyDADMA X, and (**b**) ionic conductivity of PEDOT:PolyDADMAX.

The ionic conductivity of the PolyDADMA X polyelectrolytes was investigated by EIS between 25 and 90 °C. The values are plotted in Figure 4a, which shows that PolyDADMA CF_3_SO_3_ yields the highest ionic conductivity, 2·× 10^−6^ S cm^−1^ at room temperature. The value for PolyDADMA FSI (1·× 10^−7^ S cm^−1^ at 70 °C) is consistent with a previous study (2 × 10^−7^ S cm^−1^ at 70 °C) [31], as presented in Table 1. The ionic conductivity for PolyDADMA TFSI is similar to that of PolyDADMA FSI (1·× 10^−7^ S cm^−1^ at 70 °C), as reported by Fdz. de Añastro et al. [27]. Surprisingly, the conductivity values of PolyDADMA CF_3_SO_3_ were higher than PolyDADMA FSI and PolyDADMA TFSI in all the ranges of temperatures, despite showing a higher *T*_g_. Static 19F-NMR lineshape analysis was used to study the ion dynamics of PolyDADMA CF_3_SO_3_ and PolyDADMA TFSI between 20 and 80 °C (see Figure S7), showing [CF_3_SO_3_]^−^ narrower peaks, thus suggesting a higher mobility of the anion than that of TFSI.

Finally, PolyDADMA Tos yielded the lowest ionic conductivity (2·× 10^−8^ S cm^−1^ at 70 °C), due to the large and rigid nature of the anion, likely resulting in lower mobility. This material also had the narrowest tan delta in contrast to the other three systems, which showed broad peaks reflecting more dynamic *T*_g_ [34].

Table 1 shows the obtained ionic conductivity values and activation energies (E_a_) of the polyDADMA-X polyelectrolyte series, compared to reported conductivity values. The E_a_ indicates the relative ease for the process to occur, where the limiting process here is in the physical separation of the anion from the polymeric cation backbone. Similar E_a_ values were obtained for the FSI, TFSI and CF_3_SO_3_ compounds (25.9 ± 0.4, 25.8 ± 2.5 and 29.2 ± 1.3 KJ mol^−1^, respectively) with a relatively lower activation energy for PolyDADMA Tos (13.6 ± 0.4 KJ mol^−1^). These results can be explained to some extent by taking the size and dynamics of the anion into account. The size of the anions follows: CF_3_SO_3_ (80 Å3) < FSI (95 Å3) < Tos (134 Å3) < TFSI (147 Å3) [35,36,37]. The highest E_a_ was reported for the polyDADMA CF_3_SO_3_ polyelectrolyte; as CF_3_SO_3,_ is the smallest anion, it can be better packed within the lattice, particularly compared with FSI and TFSI anions. With increasing temperature the local dynamics increase and facilitate the anion motion. Although tosylate is not the biggest anion among the series, it provides the lowest E_a_. If we consider the modulus data that shows a rather rigid polymer below *T*_g_ for this system, it could be that this flat π-conjugated anion is relatively immobile even at higher temperatures, which then presents as a lower E_a_.

### 3.2. PEDOT:PolyDADMA X

**Figure 5:**
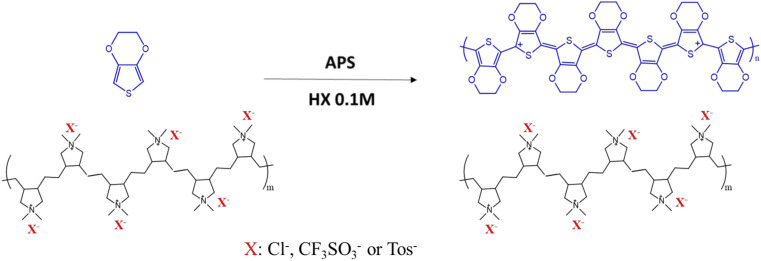
Synthesis of PEDOT:PolyDADMA X.

In order to provide electronic conductivity to PolyDADMX X systems and obtain new mixed ionic-electronic conductors, EDOT was polymerized in the presence of PolyDADMA X (Figure 5), to obtain blends of a conducting polymer (PEDOT) and polyelectrolyte (PolyDADMA X). In the case of FSI and TFSI anions, a precipitation of the product was carried out as explained above, by the addition of FSI or TFSI salt to the PEDOT:PolyDADMAC. The solubility of these compounds depends totally on the anion, which ranges from non-polar such as acetonitrile, acetone or tetrahydrofurane (in the case of FSI and TFSI), to polar such as methanol or water (Cl, CF_3_SO_3_ and Tos).

#### 3.2.1. Thermal Analysis

The TGA curves of the PEDOT:PolyDADMA X polyelectrolytes, with the anion being either Cl, FSI, TFSI, CF_3_SO_3_ or Tos, are shown in Figure 3b. PEDOT:PolyDADMA Cl, PEDOT:PolyDADMA CF_3_SO_3_ and PEDOT:PolyDADMA Tos show a two-step decomposition profile, while PEDOT:PolyDADMA FSI and PEDOT:PolyDADMA TFSI show a single step decomposition. The different decomposition temperatures could be related to an immiscibility of the blend, whose components (PEDOT and PolyDADMA X) have very different decomposition temperatures [38]. The lower decomposition step, at around 300 °C, is related to PEDOT, while the step between 350 and 450 °C is attributed to the decomposition of PolyDADMA X. Previous studies have reported that PEDOT:Cl, PEDOT:CF_3_SO_3_ and PEDOT:Tos decompose below 350 °C [39,40], while decomposition of PolyDADMA X occurs around 400 °C. In the case of PEDOT:PolyDADMA Cl, 2 peaks were observed in the TGA curve with a shoulder in the first peak. This shoulder is due to PolyDADMA Cl, which normally presents a two-step decomposition profile, at 300 °C and 450 °C [41], the first peak coinciding with the decomposition of PEDOT:Cl. This multiple-step decomposition behavior explains why PEDOT:PolyDADMA FSI and PEDOT:PolyDADMA TFSI can form a coating via solvent casting, as they are the only miscible blends.

In any case, comparison of the neat PolyDADMA X and PEDOT X (Table 2) shows that the decomposition temperatures have generally diminished in the blends. This is most likely due to interruption of the interactions within PolyDADMA X by the presence of the other cation (PEDOT) that can also interact with X. The first decomposition temperature of PEDOT:PolyDADMA X is likely related to PEDOT X.

#### 3.2.2. Ionic Conductivity

The ionic conductivity of the mixed conductors was investigated by EIS between 25 to 90 °C (Figure 4b). In all cases, a closed semicircle typical of mixed ionic electronic conductors was observed in the Nyquist plots [2]. Compared to neat PolyDADMA X, the ionic conductivity of PEDOT:PolyDADMA X increased. At 70 °C the highest ionic conductivity value was reached by PEDOT:PolyDADMA TFSI (3·× 10^−5^ S cm^−1^) followed by FSI (5·× 10^−6^ S cm^−1^), CF_3_SO_3_ (7·× 10^−7^ S cm^−1^), Tos (5·× 10^−7^ S cm^−1^) and finally Cl (2·× 10^−8^ S cm^−1^). As discussed above, the packing ability of the anion appears to be crucial. The smaller the anion, the stronger the interactions with the polymer, while the bigger anions have lower mobility. The TFSI anion appears to offer the most ideal compromise, in terms of enhancing the ionic conductivity. 

The activation energies are also influenced by the interactions between the anion and the polymers. The Tos anion resulted in the lowest E_a_ (4.0 ± 0.4 KJ mol^−1^) with FSI, TFSI, Cl and CF_3_SO_3_ having slightly higher values (8.4 ± 0.4, 8.6 ± 0.5, 8.9 ± 1.0 and 7.5 ± 1.0 KJ mol^−1^, respectively). The activation energies of the mixed conductors are much lower than that of the neat PolyDADMA X, probably because of the interaction of the anion with the other cation (PEDOT), resulting in weaker bonds between X and the cations, as discussed in the thermal stability section. These interactions are also likely to be responsible for the increased ionic conductivity of PEDOT:PolyDADMA X versus the neat PolyDADMA X. 

#### 3.2.3. Electronic Conductivity

The electronic conductivity of PEDOT:PolyDADMA FSI and PEDOT:PolyDADMA TFSI coatings was measured (Table 3), however films of PEDOT:PolyDADMA CF_3_SO_3_, PEDOT:PolyDADMA Tos and PEDOT:PolyDADMA Cl could not be cast, as discussed above.

Higher electronic conductivity values (σ_electronic_) were measured for coatings compared with pellets, since the coatings are smoother and much thinner than the pellets. The effect of coating thickness is well known in the field of electronic conductors [14]. The conductivity of PEDOT comes from the existence of polarons and bipolarons formed in the chains, where the charges can flow [42]. The FSI system results in higher electronic conductivity than the TFSI system, with conductivity values of 0.6 S cm^−1^ versus 0.25 S cm^−1^, respectively, which may be the result of a greater formation of polarons in the FSI system.

For the pellets, the highest electronic conductivity was attained by the CF_3_SO_3_ system (0.3 S cm^−1^) followed by Cl (0.1 S cm^−1^) and Tos (0.1 S cm^−1^), in contrast to FSI (0.04 S cm^−1^) and TFSI (0.02 S cm^−1^). PEDOT:Cl and PEDOT:Tos have been previously synthesized by vapor phase polymerization, showing electronic conductivity values of 400 S cm^−1^ and 700 S cm^−1^, respectively [17]. The tosylate anion can provide π -cojugated electrons where charges of PEDOT can be delocalized. The doping effect of acids on PEDOT has been well studied [43], and TFMSA, which was involved in the polymerization of PEDOT:PolyDADMA CF_3_SO_3_, is known as a superacid. These factors make FSI and TFSI poorer dopants in contrast to Cl, CF_3_SO_3_ and Tos.

Although these electronic conductivity (σ_electronic_) values do not seem high, even in thick pellet form, the values are close to those of the commercially available Clevios PH1000 (0.2 S cm^−1^).

### 3.3. Neat OIPC

In order to enhance the mechanical properties and ionic conductivity, OIPCs with the same anions used above, were synthesized to prepare composites of 80/20 wt% PEDOT:PolyDADMA X/[C_2_mpyr][X]. Previous studies have indicated that the electronic conductivity can be improved by doping conducting polymers with ILs [22,44]. In order to understand the effects of the OIPCs on the composite systems, prior thermal and conductivity characterization was performed and is presented and discussed below.

#### 3.3.1. Thermal Analysis

**Figure 6:**
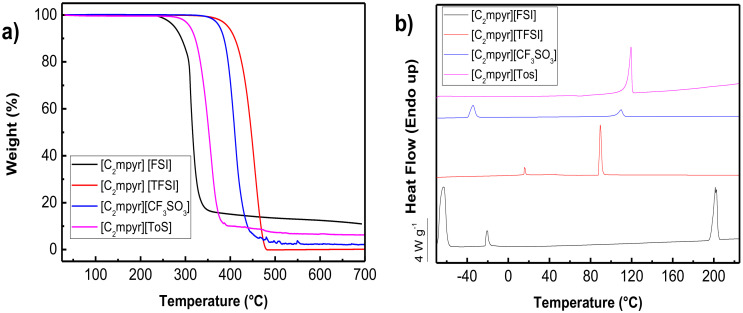
(**a**) TGA and (**b**) DSC curves of the organic ionic plastic crystals (OIPCs) with different anions.

The TFSI anion provides the highest thermal stability, with one-step decomposition observed at 454 °C (Figure 6a). The *T*_d_ of [C_2_mpyr][FSI] was measured to be at 313 °C, which is close to that previously reported by Yamada et al. (300 °C) [45]. The decomposition temperature of [C_2_mpyr][TFSI] is 150 degrees higher than the corresponding FSI salt, in a similar trend as the PolyDADMA X samples (450 and 300 °C respectively). The thermogram related to the CF_3_SO_3_ OIPC presents a T_d_ around 410 °C, which is similar to that previously reported [46] for N-butyl-N-methylpyrrolidinium triflate. Finally, [C_2_mpyr][Tos] presents a *T*_d_ of 357 °C, which is in good agreement with that reported by Dhahri et al. [47] for the [C_2_mpyr] OIPC based on imidazolium.

As previously discussed in the section on the thermal stability of PolyDADMA X, the anion has a huge impact on the decomposition behavior. The T_d_ of these salts follows the same trend observed in PolyDADMA X, that is: T_d_ FSI < Tos < CF_3_SO_3_ < TFSI. All of them are thermally stable up to at least 300 °C, which enables their use in many electrochemical applications.

Thermal analysis of the OIPCs by DSC showed different thermal behaviors depending on the anion. OIPCs are characterized by having one or more solid-solid transition temperatures prior to melting, a long-range ordered crystalline lattice and entropies of fusion following the Timmerman’s criterion (<20 JK^−1^mol^−1^) [48]. The existence of solid-solid phase transition temperatures, T_S-SS_, is accompanied by an increase of the ions’ mobility due to rotational motions and/or major structural reorientation of the ions, resulting in a disordered lattice [49]. The solid-solid phase transitions also often result in a small entropy of fusion that gives the material “plastic–crystal” properties.

The DSC curves are presented in Figure 6b and summarized in Table S1. OIPCs with the FSI and TFSI salts present solid-solid phase transitions with similar values to previous studies [23,50]. In the case of [C_2_mpyr][FSI], there are two T_S-SS_ at −64 and −21 °C and a melting point at 201 °C with a low entropy value (10.1 J K^−1^ mol^−1^). On the other hand, [C_2_mpyr][TFSI] presents two T_S-SS_ occurring at 16 and 46 °C before the melting point at 90 °C with a higher entropy (29.3 J K^−1^ mol^−1^). The higher range of temperatures between the T_S-S_ and melting point and the lower entropy of fusion explains the higher plasticity of [C_2_mpyr][FSI] versus [C_2_mpyr][TFSI] [49].

The thermal behavior of [C_2_mpyr][CF_3_SO_3_] is characterized by having one Ts-s at −34.4 °C and a melting point at 110 °C. Additionally, the entropy of fusion of this salt satisfies the Timmerman’s criterion presenting 12.5 J K^−1^ mol^−1^ confirming this material to be an OIPC with a plastic behavior similar to [C_2_mpyr][FSI] and over a significant range of temperature. In contrast, [C_2_mpyr][Tos] does not present any solid-solid phase transitions and has a broad melting point at 120 °C with an entropy of 67.5 J K^−1^ mol^−1^. These values are close to those previously reported in the literature, as listed in Table S1. The high value of entropy and absence of a Ts-s means that this salt cannot be considered to have plastic crystal behavior.

#### 3.3.2. Ionic Conductivity

**Figure 7:**
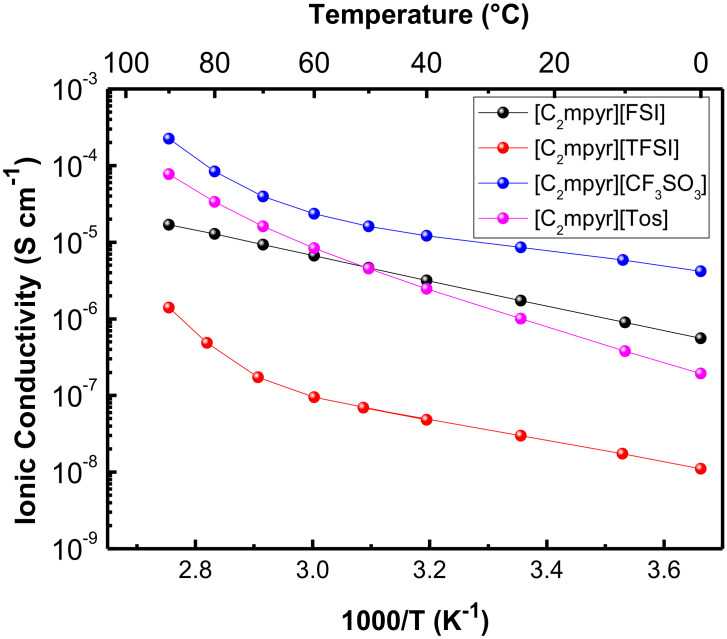
Ionic conductivity of OIPCs with different anions.

Figure 7 compares the ionic conductivity of the different salts. In all cases, the conductivity increased with higher temperature, following typical Arrhenius behavior. In the cases of TFSI, CF_3_SO_3_ and Tos, a dramatic increase of conductivity is observed at high temperatures as the trend is influenced by the melting point of the compounds near 100 °C.

The ionic conductivity values are intimately related to the thermal behavior of the materials. As previously discussed for OIPCs, the existence of a Ts-s and melting point with low entropy, are typical of OIPCs with significant ion dynamics. The maximum ionic conductivity value at 70 °C was 4·× 10−5 S cm−1, for [C2mpyr][CF3SO3]. This can be attributed to the fact that the CF3SO3 anion is the smallest of the anion series, along with the thermal behavior discussed above. On the other hand, [C2mpyr][TFSI] presented the lowest ionic conductivity values (2·× 10−7 S cm−1 at 70 °C), close to those previously reported in the literature [51]. The [C2mpyr][FSI] shows an ionic conductivity value of 9·× 10−6 S cm−1 at 70 °C, which is slightly different from that previously reported (6·× 10−6 S cm−1 at 70 °C) [52], but the difference could be attributed to different geometries of the EIS cell or differences in the measurement procedure. Surprisingly, given the absence of OIPC behavior, the values related to [C2mpyr][Tos] (2·× 10−5 S cm−1 at 70 °C) are close to [C2mpyr][FSI] although the latter has a higher conductivity at lower temperatures. Also, very surprisingly, [C2mpyr][Tos] presents higher ionic conductivity values than [C2mpyr][TFSI] across the entire temperature range in this study. This possibly could be related to the fact that some small impurity is present in the[C2mpyr][Tos], as suggested by the broad peak onset of the melting point. This is typical of liquidus like behavior where, even a fractional impurity can lead to a liquid eutectic at the grain boundaries of the pure [C2mpyr][Tos] crystals. This liquid phase would increase as the final melt is approached and lead to an increase in high ionic conductivity. Previous studies have shown that even a 1% impurity (or dopant) component can increase the ionic conductivity dramatically [53,54].

The activation energies for conductivity of these salts are quite low but this is the case mainly for the ones classified as an OIPC: [C_2_mpyr][FSI] (12.9 ± 0.1 KJ mol^−1^), [C_2_mpyr][TFSI] (11.42 ± 0.3 KJ mol^−1^) and [C_2_mpyr][CF_3_SO_3_] (8.5 KJ ± 0.2 mol^−1^). The more disordered a compound is, the lower the activation energy is [55], and this disorder is connected to the properties of the OIPC, the Ts-s and low entropy of fusion. [C_2_mpyr][CF_3_SO_3_], which has a large temperature range between the T_S-S_ and the T_m_, a low melting point as well as a low melting entropy, was measured to have the lowest activation energy.

### 3.4. PEDOT:PolyDADMA X + OIPC

**Figure 8:**
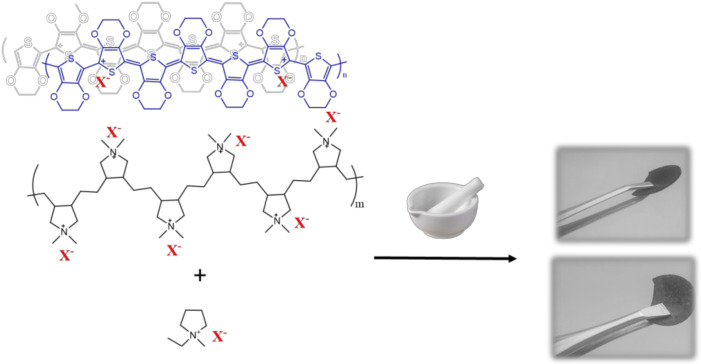
Preparation of 80/20 PEDOT:PolyDADMA X / C_2_mpyr X composites.

Finally, the synthesized OIPCs were added to PEDOT:PolyDADMA X blends, in order to study the doping effect of the OIPCs and improve the transport properties. Composites of 80/20 wt% PEDOT:PolyDADMA X/[C_2_mpyr][X] were prepared by dissolving the solids and mixing them in liquid state. Good coatings were formed from the FSI and TFSI system but not with the other anions, probably because of the immiscibility between polymers. SEM images of the neat PEDOT:PolyDADMA FSI and PEDOT:PolyDADMA TFSI and their composites were taken (Figure S10), where it seems the OIPC is able to cover the material forming a more continuous film. Hence, the composites were mixed in solid state with a mortar and pressed into pellets to compare them (see Figure 8).

#### 3.4.1. Electronic Conductivity

The electronic conductivity of the 80/20 PEDOT:PolyDADMA X / C_2_mpyr X pellets of ~500 µm of thickness were measured by 4PP, under the same conditions as PEDOT:PolyDADMA X. [C_2_mpyr][CF_3_SO_3_] led to a significant enhancement of the electron transport from the pure PEDOT:PolyDADMA CF_3_SO_3_ (0.3 S cm^−1^) to the value of 0.73 S cm^−1^. FSI and TFSI OIPCs slightly enhanced the conductivity from their respective values; for PEDOT:PolyDADMA X conductivity increased from 0.04 S cm^−1^ and 0.02 S cm^−1^ to 0.08 S cm^−1^ and 0.06 S cm^−1^, respectively. Finally, the electronic conductivity of the 80/20 PEDOT:PolyDADMA Tos/[C_2_mpyr][Tos] could not be measured, possibly because of the apparent hygroscopic behavior of the material and/or a very low conductivity value.

#### 3.4.2. Ionic Conductivity

**Figure 9:**
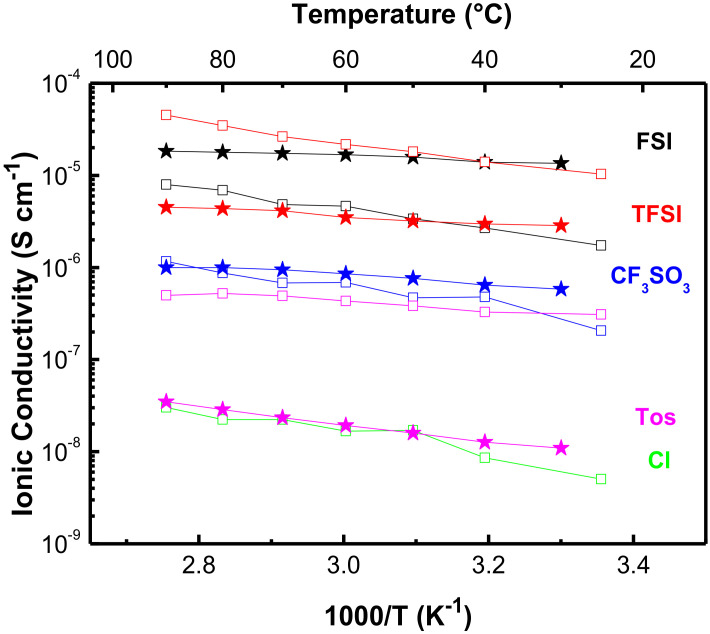
Ionic conductivity values of PEDOT:PolyDADMA X (squares) and 80/20 PEDOT:PolyDADMA X / [C_2_mpyr][X] composites (stars), where X is FSI (black), TFSI (red), CF_3_SO_3_ (blue), Tos (pink) and Cl (green).

EIS measurements of the composites were carried out between 30 and 90 °C. The typical closed semicircle of MIECs was observed in all the samples. Evaluation of the ionic conductivity of the 80/20 wt% PEDOT:PolyDADMA X/[C_2_mpyr][X] (Figure 9) shows there is a significant increase of one order of magnitude for the FSI (2·× 10^−5^ S cm^−1^ at 70 °C) composite versus PEDOT:PolyDADMA FSI with no OIPC (5·× 10^−6^ S cm^−1^ at 70 °C). The ionic conductivity value of PEDOT:PolyDADMA CF_3_SO_3_ at 70 °C was enhanced from 7·× 10^−^^7^ S cm^−^^1^ to 1·× 10^−^^6^ S cm^−^^1^ with addition of the OIPC. Finally, in the case of TFSI and Tos, the ionic conductivity value decreased from 3·× 10^−^^5^ S cm^−^^1^ and 5·× 10^−^^7^ to 4·× 10^−^^6^ S cm^−^^1^ and 2·× 10^−^^8^ S cm^−^^1^, respectively. The increase of ionic conductivity from the addition of [C_2_mpyr][FSI] and [C_2_mpyr][CF_3_SO_3_] could be due to the fact that both of these salts have higher intrinsic ion dynamics following Timmerman’s criterion (see Section 3.3.1.) for plastic crystals and hence considered as OIPCs and these higher dynamics are retained in the composites. On the other hand, composites formed with the salts [C_2_mpyr][TFSI] and [C_2_mpyr][Tos] do not fit into this criterion and have intrinsically lower ion dynamics as seen from the conductivity of the pure salts (Figure 7); hence their presence in the composite decreases the overall ionic conductivity,

The Ea of the composites also changed with the addition of the OIPCs. In general, the incorporation of [C2mpyr][X] into the system decreased the activation energy from 8.4 KJ mol−1 (PEDOT:PolyDADMA FSI), 8.6 KJ mol−1 (PEDOT:PolyDADMA TFSI), 7.5 KJ mol−1 (PEDOT:PolyDADMA CF3SO3) to 2.3 KJ mol−1, 3.4 KJ mol−1 and 3.6 KJ mol−1, respectively (Table 4). Interestingly, the Ea of the Tos composite was increased from 4 KJ mol−1 (PEDOT:PolyDADMA Tos) to 8.7 KJ mol−1 (80/20 PEDOT:PolyDADMA Tos/[C2mpyr][Tos]). This contrast could be due to the absence of plastic crystal behavior in [C2mpyr][Tos].

#### 3.4.3. Cyclic Voltammetry

**Figure 10:**
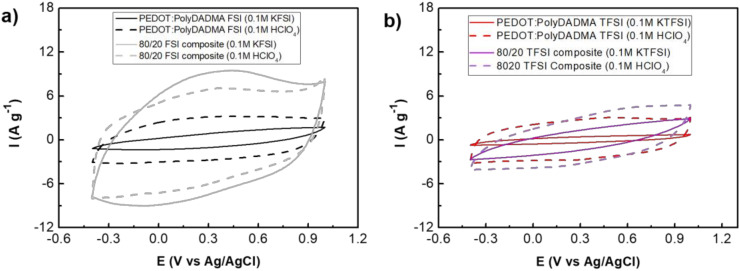
Voltammograms at 0.2 V s^−1^ of (**a**) PEDOT:PolyDADMA FSI (black lines) and 80/20 PEDOT:PolyDADMA FSI / [C_2_mpyr][FSI] (grey lines) using 0.1 M KFSI (solid lines) and 0.1 M HClO_4_ (dashed lines) and (**b**) PEDOT:PolyDADMA TFSI (red lines) and 80/20 PEDOT:PolyDADMA TFSI / [C_2_mpyr][TFSI] (purple lines) using 0.1 M KTFSI (solid lines) and 0.1 M HClO_4_ (dashed lines). KFSI = Potassium bis(fluorosulfonyl)imide.

The electrochemical behavior was only analyzed for the FSI and TFSI systems since the other systems were unable to form a film, as discussed above. The typical rectangular shape of capacitive behavior was observed, as shown in Figure 10. 

Different aqueous media were used as electrolytes, including 0.1 M KFSI and 0.1 M KTFSI as neutral electrolytes with the same anion as the system to avoid anion exchange and possible secondary reactions, and 0.1 M HClO_4_ was used to observe the proton doping effect on PEDOT. The potential window was cycled from −0.4 V to 1 V vs. Ag/AgCl.

In general, the FSI system shows a much higher capacity than TFSI since the electronic conductivity is higher. Focusing on the FSI system, the voltammogram of PEDOT:PolyDADMA FSI in 0.1 M KFSI is quite resistive, unlike when 0.1 M HClO_4_ was used as the electrolyte, because of the effect of proton doping on PEDOT as discussed above. The capacity was even higher when [C_2_mpyr][FSI] was added to obtain 80/20 PEDOT:PolyDADMA FSI / [C_2_mpyr][FSI]; this indicated a more efficient doping was achieved for PEDOT. When the 80/20 FSI composite was cycled using 0.1 M HClO_4_ as the electrolyte, the capacity was diminished compared to the voltammogram obtained using 0.1 M KFSI as the electrolyte.

Although TFSI is much more resistive than FSI, a similar behavior is observed in the voltammograms. PEDOT:PolyDADMA TFSI undergoes doping with protons when HClO_4_ is used as the electrolyte. The 80/20 TFSI composite also shows an enhanced doping effect when 0.1 M KTFSI was used, compared with the neat PEDOT:PolyDADMA TFSI. However, in this case the [C_2_mpyr][TFSI] is not as effective as [C_2_mpyr][FSI]. Finally, when 0.1 M HClO_4_ was used as the electrolyte, the proton doping and OIPC doping were additive. This doping effect of organic salts have been observed previously [56]. 

## 4. Conclusions

Blends of PEDOT and PolyDADMA X were obtained as mixed ionic-electronic conductors, using composites of novel polyelectrolytes and OIPCs. A series of PolyDADMA X polyelectrolytes were prepared, where X was either chloride (Cl), bis(fluorosulfonyl)imide (FSI), bis(trifluoromethylsulfonyl)imide (TFSI), triflate (CF3SO3) or tosylate (Tos). Coatings could only be formed when X was FSI or TFSI, as a result of a possible immiscibility between the polymers, as indicated by the thermal analysis. The maximum electronic conductivity was obtained for the PEDOT:PolyDADMA FSI coating (0.6 S cm−1), with an ionic conductivity value of 5·10-6 S cm−1 at 70 °C. The PEDOT:PolyDADMA TFSI coating showed a lower electronic conductivity (0.25 S cm−1) with an ionic conductivity of 4·10-6 S cm−1 at 70 °C. CF3SO3 has been postulated as a good anion with promising results, reaching the highest electronic conductivity value (0.3 S cm−1) among the PEDOT:PolyDADMA X pellets, the highest ionic conductivity value among the different OIPCs (4·× 10−5 S cm−1 at 70 °C) and having a higher ionic conductivity value than the FSI and TFSI samples as a polyelectrolyte (PolyDADMA CF3SO3) (1·× 10−6 S cm−1 at 70 °C).

Finally, 80/20 PEDOT:PolyDADMA X / [C2mpyr][X] composites were prepared and their ionic conductivity and electrochemical capacity were measured. Interestingly, the most promising results were achieved by the authentic OIPCs; [C2mpyr][FSI] and [C2mpyr][CF3SO3]. The new OIPC [C2mpyr][CF3SO3] exhibited an important electronic transport boost from the neat PEDOT:PolyDADMA CF3SO3 (0.30 S cm−1) to the value of 0.73 S cm−1. Nonetheless, both OIPCs enhanced the ionic conductivity of PEDOT:PolyDADMA X, with a bigger impact in the case of [C2mpyr][FSI]. A clear doping effect was evident for the FSI OIPC, in terms of the electrochemical performance.

Whilst systems based on PEDOT:PSS usually have higher electronic conductivity it is important to recognize that they are normally characterized by the presence of around 20% of water or even more in the material [12,57]. The incorporation of different polyDADMA X polymers in the PEDOT X blends shows the potential of using such ionic polymers to tune the material’s properties with respect to ionic and electronic conductivities as well as mechanical properties in the absence of water. This research also highlights the benefits of incorporating OIPCs as dopants in conducting polymers and mixed ionic-electronic conductors, and demonstrates the potential of the new compounds presented herein (PolyDADMA CF3SO3, PolyDADMA Tos, [C2mpyr][CF3SO3] and [C2mpyr][Tos]) for application in electrochemical storage systems.

## Figures and Tables

**Table 1:** Ionic conductivities (σ_ionic_) of the PolyDADMA X polyelectrolytes obtained experimentally at 70 °C, compared to literature values and the activation energy (E_a_).

Compound	Literature Values	Experimental Values	
	*σ_ionic_*/S cm^−1^	*σ_ionic_*/S cm^−1^	*E_a_*/KJ mol^−1^
PolyDADMA FSI	2·× 10^−7^ [31]	1·× 10^−7^	25.9 ± 0.4
PolyDADMA TFSI	1·× 10^−7^ [27]	-	25.8 ± 2.5 [27]
PolyDADMA CF_3_SO_3_	-	1·× 10^−6^	29.2 ± 1.3
PolyDADMA Tos	-	2·× 10^−8^	13.6 ± 0.4

**Table 2:** Decomposition temperature of PEDOT:X, PolyDADMA X and PEDOT:PolyDADMA X, where T_1, d_ means the first step of decomposition and T_2, d_ means the second step of decomposition.

X	*T_d PEDOT:X_*/°C	*T_d PolyDADMA X_*/°C	*T_1, d PEDOT:PolyDADMA X_*/°C	*T_2, d PEDOT:PolyDADMA X_*/°C
Cl	300 [39]	300 and 450 [41]	281, 295	378
FSI	-	300 [31]	-	340
TFSI	-	450 [31]	-	423
CF_3_SO_3_	360 [40]	427	301	345
Tos	343 [40]	382	296	343

**Table 3:** Electronic conductivity (σ_electronic_) of coatings and pellets of the PEDOT:PolyDADMA X samples with varying anions.

	Coating		Pellet	
	Thickness/μm	*σ_electronic_*/S cm^−1^	Thickness/μm	*σ_electronic_*/S cm^−1^
FSI	70	0.60	400	0.04
TFSI	75	0.25	500	0.02
Cl	-	-	250	0.10
CF_3_SO_3_	-	-	350	0.30
Tos	-	-	250	0.10

**Table 4:** Ionic conductivity values and E_a_ of PEDOT:PolyDADMA X and 80/20 wt% composites at 50 °C.

X	PEDOT:PolyDADMA X		80/20 wt% PEDOT:PolyDADMA X/C_2_mpyr X	
	σ_ionic_/S cm^−1^	*E_a_*/KJ mol^−1^	σ_ionic_/S cm^−1^	*E_a_*/KJ mol^−1^
FSI	5·10^−6^	8.4	2·10^−5^	2.3 ± 0.2
TFSI	3·10^−5^	8.6	4·10^−6^	3.4 ± 0.2
Cl	2·10^−8^	8.9	-	-
CF_3_SO_3_	7·10^−7^	7.5	1·10^−6^	3.6 ± 0.3
Tos	5·10^−7^	4.0	2·10^−8^	8.7 ± 0.4

## Supplementary Materials

The following are available online at https://www.mdpi.com/2073-4360/12/9/1981/s1. Figure S1. Dynamic mechanical analysis (DMA) curves of the neat PolyDADMA FSI, TFSI, CF_3_SO_3_ and Tos polyelectrolytes; Figure S2. 1H-NMR spectrum of [C_2_mpyr][Tos] in D_2_O; Figure S3. 1H-NMR of [C_2_mpyr][CF_3_SO_3_] with 4-(Trifluoromethyl) benzaldehyde in DMSO; Figure S4. 19F-NMR of [C_2_mpyr][CF_3_SO_3_] with 4-(Trifluoromethyl) benzaldehyde in DMSO; Figure S5. 1H-NMR of PolyDADMA Tos in D_2_O; Figure S6. 1H-NMR of PolyDADMA CF_3_SO_3_ with 4-(Trifluoromethyl) benzaldehyde in DMSO; Figure S7. 19F-NMR of PolyDADMA CF_3_SO_3_ with 4-(Trifluoromethyl) benzaldehyde in DMSO; Figure S8. Static 19F-NMR of PolyDADMA TFSI and PolyDADMA CF_3_SO_3_ at different temperatures; Figure S9. MAS spectra of PolyDADMA TFSI and PolyDADMA CF_3_SO_3_; Figure 10. SEM images of PEDOT:PolyDADMA FSI, TFSI and their composites with [C_2_mpyr][FSI] and [C_2_mpyr][TFSI] respectively; Table S1. Thermal properties of the OIPCs from DSC analysis, compared to the literature values.
