# Supplementary material for: Mixed Ionic-Electronic Conductors Based on PEDOT:PolyDADMA and Organic Ionic Plastic Crystals
Source: Polymers (Basel). 2020 Aug 31;12(9):1981. doi: 10.3390/polym12091981 (PMC7563752; doi:10.3390/polym12091981)
Supplement: Supplementary file 1 [file polymers-12-01981-s001.pdf]

Supporting Information

Mixed Ionic-Electronic Conductors based on PEDOT:PolyDADMA and Organic Ionic Plastic Crystals

Rafael Del Olmo [1], Nerea Casado [1,*], Jorge L. Olmedo-Martínez [1], Xiaoen Wang [2] and Maria Forsyth [1,2,3,4,*]

[1] POLYMAT University of the Basque Country UPV/EHU, Joxe Mari Korta Center, Avda. Tolosa 72, 20018 Donostia-San Sebastian, Spain; rafael.delolmo@polymat.eu (R.D.O.); jolmedo001@ikasle.ehu.es (J.L.O.-M.)

[2] Institute for Frontier Materials (IFM), Deakin University, Geelong, VIC 3217, Australia; xiaoen.wang@deakin.edu.au

[3] IKERBASQUE, Basque Foundation for Science, 48011 Bilbao, Spain

[4] ARC Centre of Excellence for Electromaterials Science (ACES), Deakin University, Burwood, VIC 3125, Australia

* Correspondence: nerea.casado@ehu.eus (N.C.); maria.forsyth@deakin.edu.au (M.F.); Tel.: +34 943018846 (N.C.); Tel.: + 61 3 92446821 (M.F.)

Received: 30 July 2020; Accepted: 29 August 2020; Published: date

1. Characterization Methods

All the compounds were characterized by thermogravimetric analysis (TGA), performed using a TGA Q-500 thermobalance, with a standard furnace coupling and nitrogen flow of 50 cm^3 min^{-1}. Sample weight was between 1 and 15 mg. Then, the decomposition temperature, T_d, was obtained from the maximum of the first derivate of the thermogram.

The organic salts were characterized by differential scanning calorimetry (DSC). The experiments were performed using a Perkin Elmer 8000 DSC equipped with an Intracooler II and calibrated with indium and tin standards. The heating rate was 10 ˚C min^{-1} in the temperature range of −70 to 225 °C and between 3 and 10 mg of sample was used every time. The measurements were performed by sealing the samples in aluminum pans. The samples were first heated from room temperature to 225 °C to erase thermal history, then cooling and finally a second heating was performed.

Compressive mode dynamic mechanical analysis (DMA, PerkinElmer DMA8000) was used to analyze the thermal behavior of the polymers when nothing was observed by DSC. The polymer was pressed in KBr die and dried at 70 ˚C under vacuum overnight to finally obtain pellets of around 2 mm of thickness and 13 mm of diameter. The temperature range of DMA was from 40 °C to 200 °C and the frequency was set at 1 Hz. The measurements were performed in a N_2 filled glovebox with a H_2O level lower than 100 ppm.

The ionic conductivities were measured by electrochemical impedance spectroscopy (EIS) using an Autolab 302N potentiostat galvanostat (Metrohm AG, Herisau, Switzerland) with the temperature controlled by a Microcell HC station. PEDOT based samples were measured making pellets of around 500 µm and 11 mm of diameter. Polyelectrolytes and organic salts were solvent cast on stainless steel electrodes. All the samples were dried at 70 °C under vacuum overnight. Afterward, the samples were sandwiched between two stainless steel electrodes (with a surface area of 0.5 cm^2). The plots were obtained by applying a 10 mV perturbation to an open circuit potential in the frequency range of 1 MHz to 0.1 Hz. Electronic conductor samples were analyzed considering the resistance of the electronic conduction negligible versus ionic resistance as previously done by McDonald et al. [1]. The activation energy (Ea) of the different materials was also studied in the linear region using the Arrhenius Equation (Eq. 1):

$$\sigma = \sigma_0 exp\left(^{-Ea}/_{RT}\right) \qquad \text{(Eq. S1)}$$

where σ is the ionic conductivity, σ_0 is the pre-exponential factor, *Ea* is the activation energy, R the universal gas constant and finally T is the absolute temperature.

The electronic sheet resistance were measured using a Jandel 4-Point Probe with the RM3000+ test unit. The samples were pellets with a thickness between 250 and 500 µm and 13 mm in diameter. Coatings with a thickness of around 50 µm were measured when they were possible to form. Electronic conductivity was calculated taking into account the thickness of the samples as follows the Eq. S2

$$\boldsymbol{\sigma_{elec.}} = \boldsymbol{R_S \cdot t} \qquad \text{(Eq. S2)}$$

where $\sigma_{elec.}$ is the electronic conductivity, R_S is the sheet resistance and *t* the thickness of the sample.

To evaluate the electrochemical performance of the system, cyclic voltammetry (CV) was carried out with an Autolab PGSTAT204 potentiostat in a conventional three electrode set up. A platinum wire was used as the counter electrode, Ag/AgCl as the reference electrode and glassy carbon as the working electrode. The samples were dissolved in the relevant solvent to finally drop cast a known quantity of material on top of the carbon electrode. The experiments were performed at 0.2 V s^{-1} .

2. Results

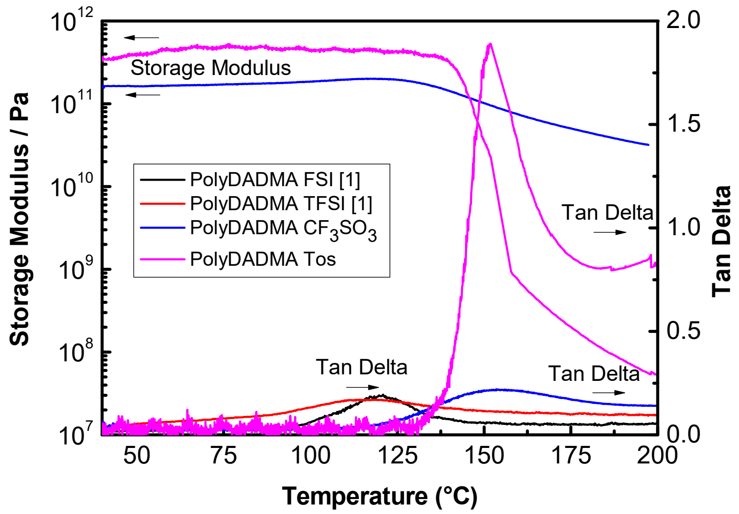

Figure S1. Dynamic mechanical analysis (DMA) curves of the neat PolyDADMA FSI, TFSI, CF_3SO_3 and Tos polyelectrolytes.

Table S1. Thermal properties of the OIPCs from DSC analysis, compared to the literature values.

	Experimental values				Literature values			
	III-II / °C	*II-I* / °C	T_m / °C	ΔS / J K^{-1} mol^{-1}	*III-II* / °C	*II-I* / °C	T_m / °C	ΔS ± 5% / J K^{-1} mol^{-1}
[C_2mpyr][FSI]	-64	-21	201	10.1	-72	-22	203 [2]	9.6
[C_2mpyr][TFSI]	16	46	90	29.3	16	40	89 [3]	27.3
[C_2mpyr][CF_3SO_3]	-	-34.4	110	12.5	-	-	-	-
[C_2mpyr][Tos]	-	-	120	67.5	-	-	120 [4]	71

For $[CF_3SO_3]^-$ compounds, an equimolar quantity of 4-(Trifluoromethyl) benzaldehyde was added as standard to quantify ^1H and ^{19}F and thus, see if the ratio between the anion and the cation is one.

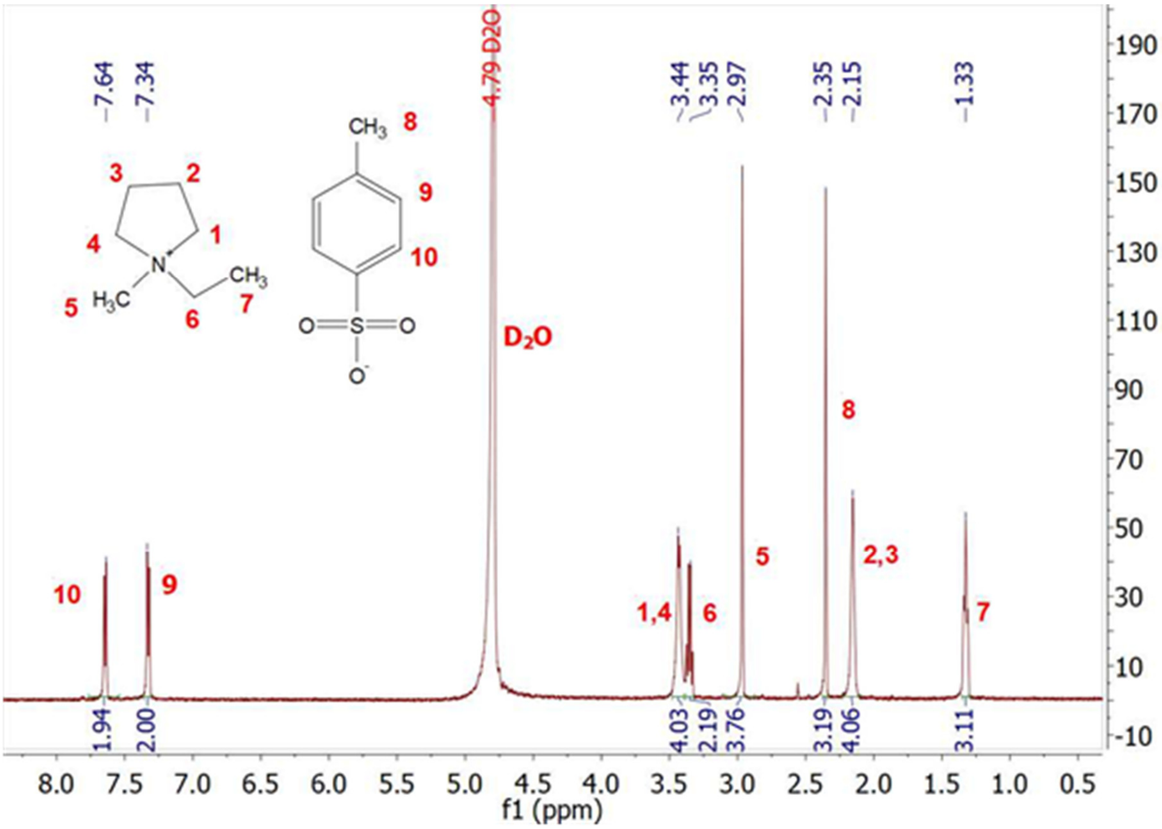

Figure S2. ^1H-NMR spectrum of $[C_2mpyr][Tos]$ in D_2O.

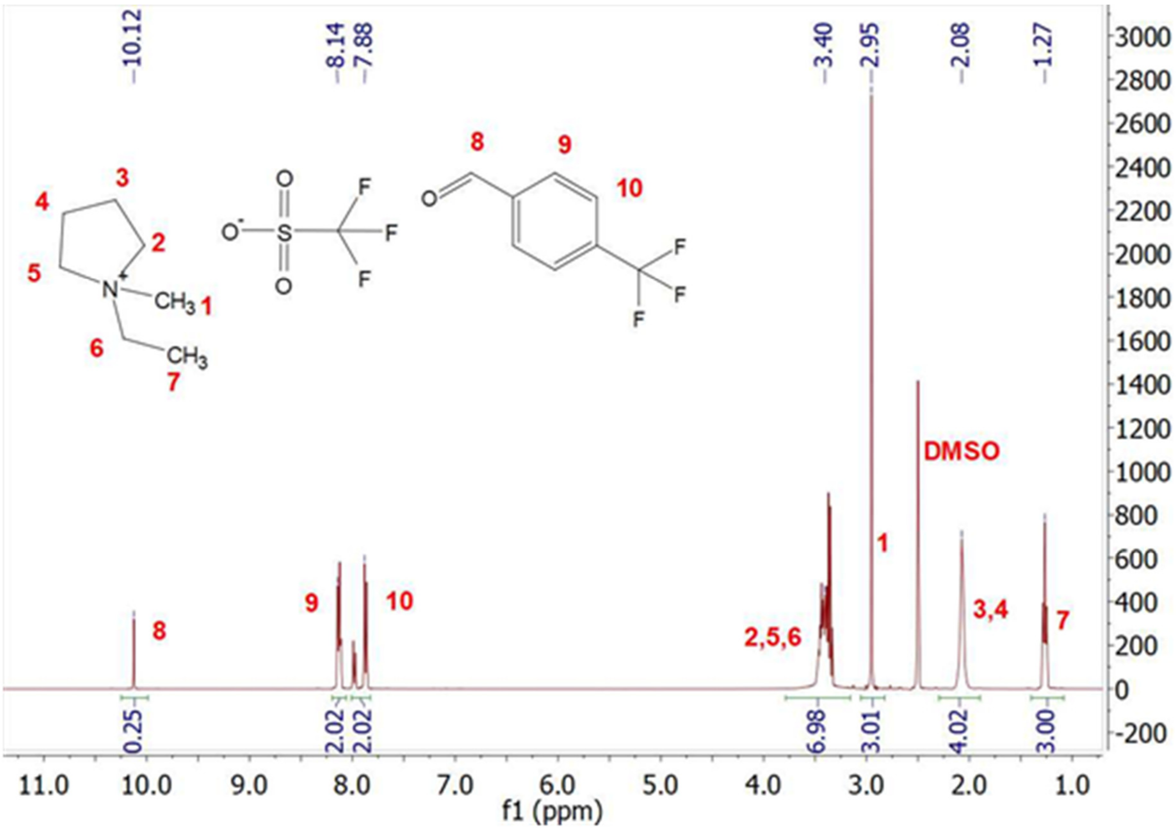

Figure S3. ^{1}H-NMR of [C2mpyr]][CF3SO3] with 4-(Trifluoromethyl)benzaldehyde in DMSO.

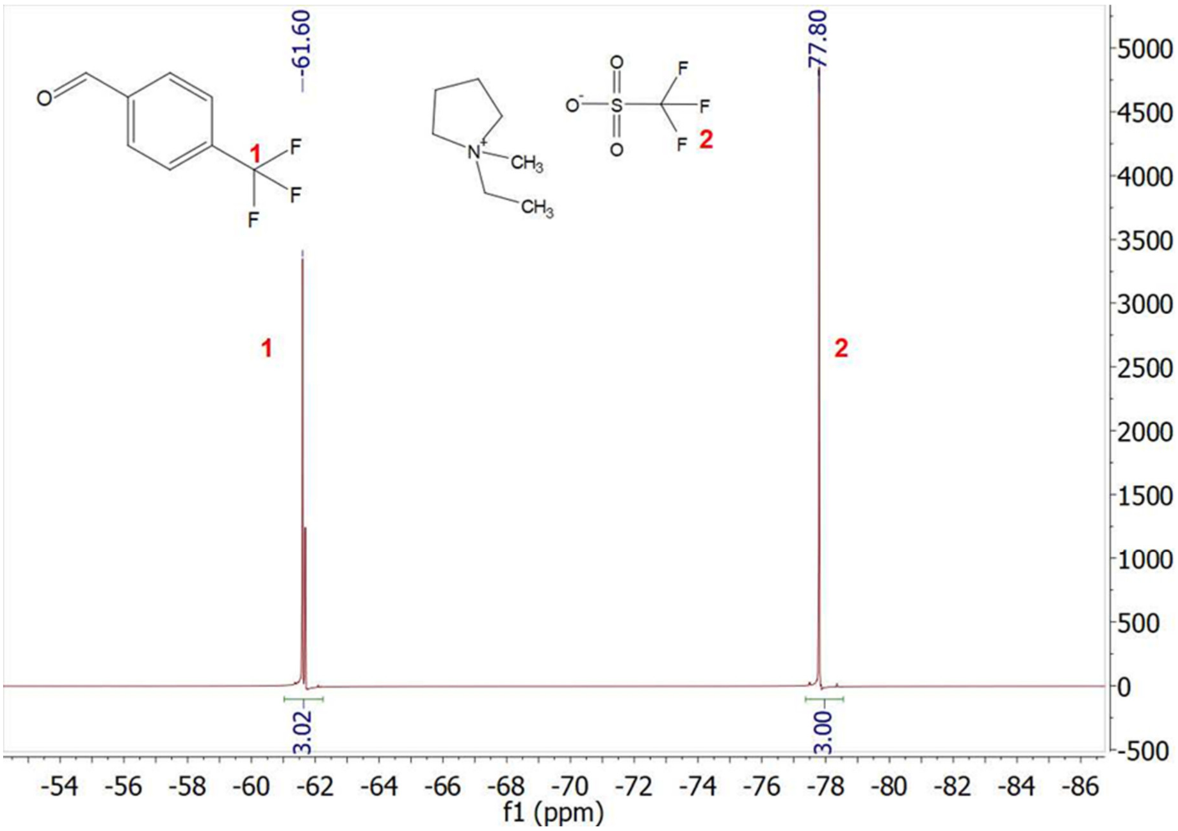

Figure. S4. 19F-NMR of [C2mpyr][CF3SO3] with 4-(Trifluoromethyl) benzaldehyde in DMSO

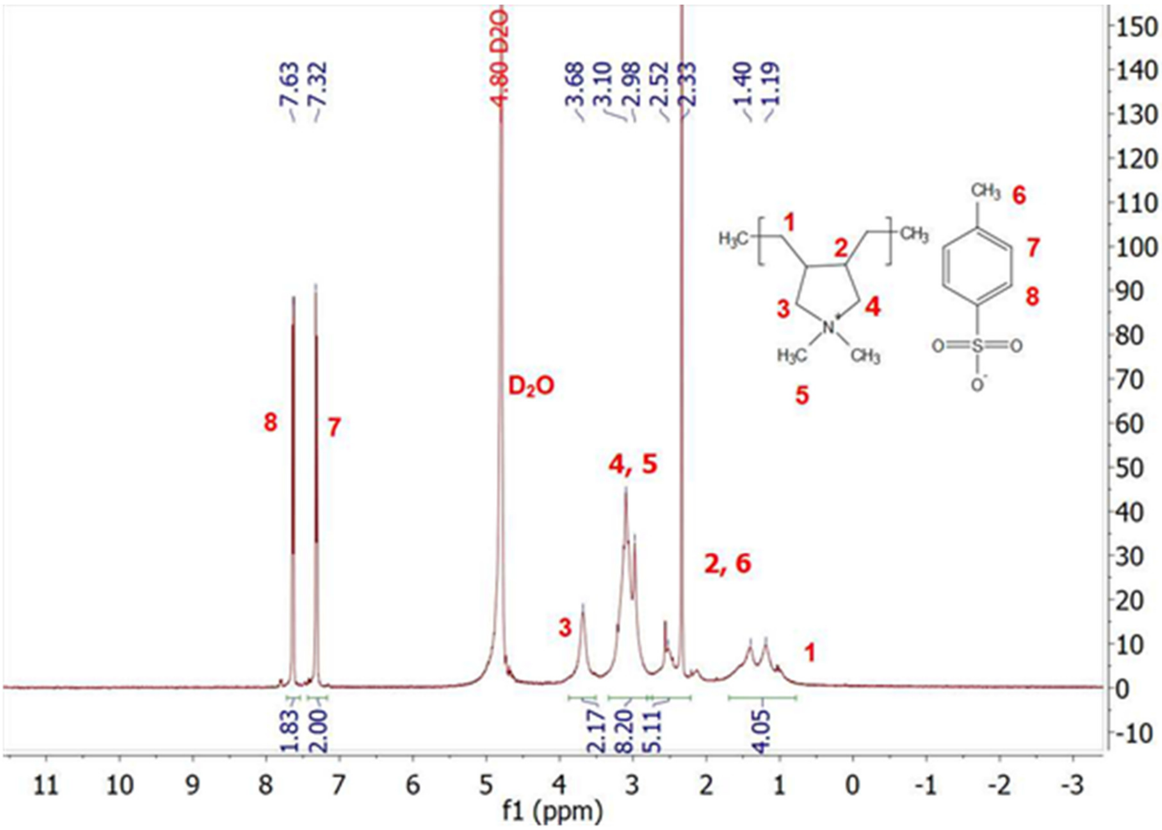

Figure S5. ^{1}H- NMR of PolyDADMA Tos in D_2O.

For PolyDADMA CF_3SO_3, 4-(Trifluoromethyl)benzaldehyde was in excess of around 1.2 over the cation and the anion. Traces of water was observed as well.

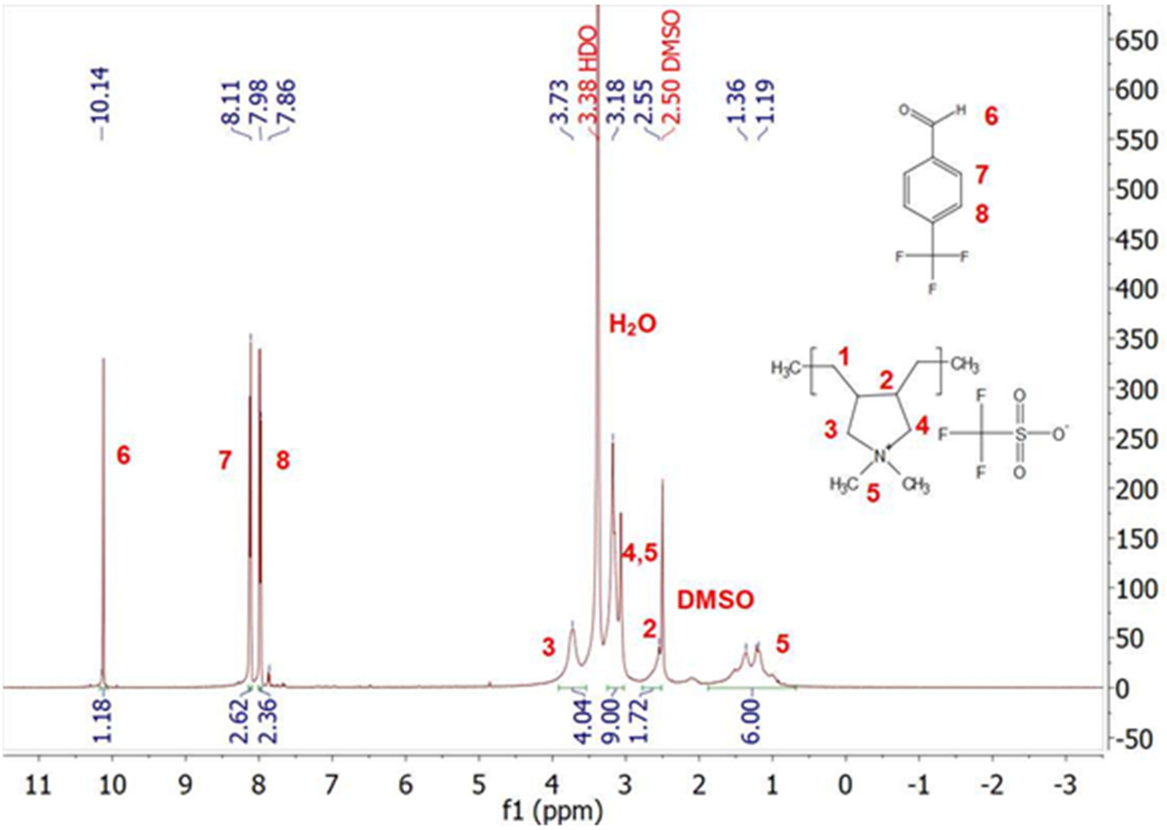

Figure S6. ^{1}H-NMR of PolyDADMA CF_3SO_3 with 4-(Trifluormethyl)benzaldehyde in DMSO.

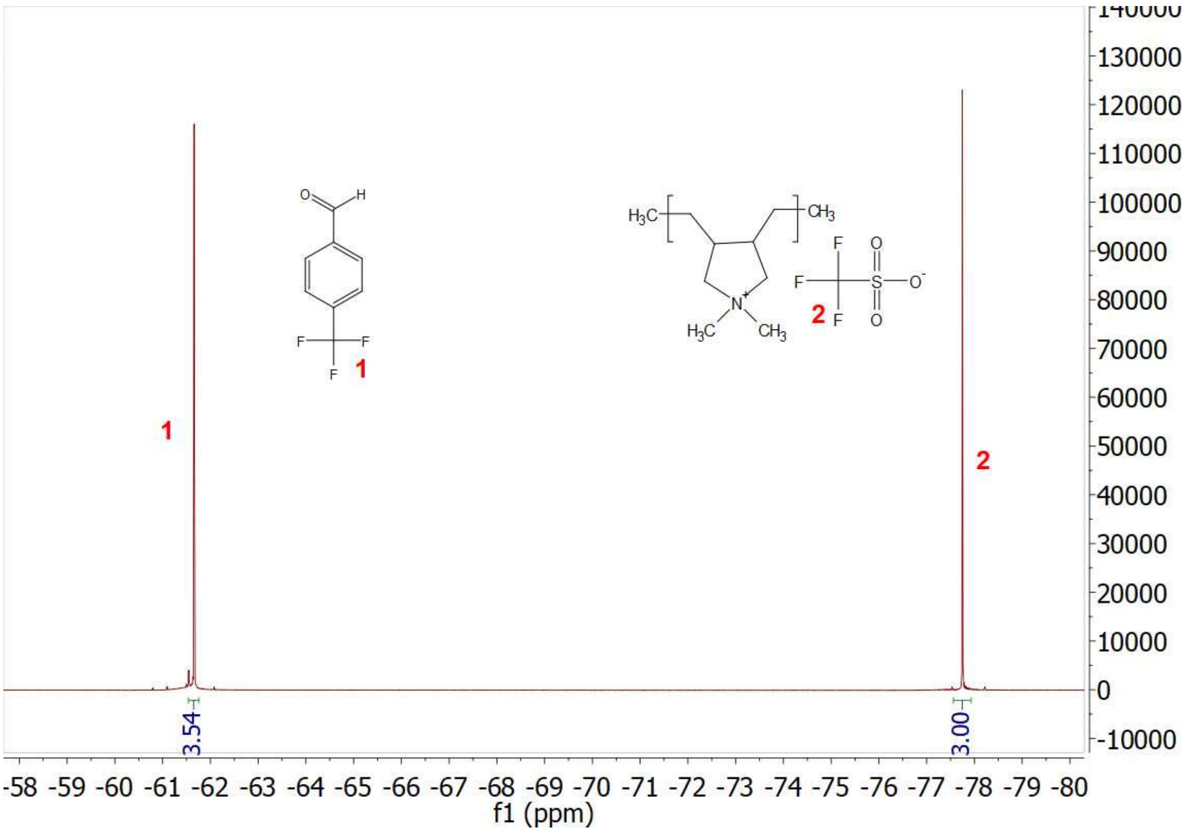

Figure S7. ^{19}F-NMR of PolyDADMA CF_3SO_3 with 4-(Trifluoromethyl)benzaldehyde in DMSO.

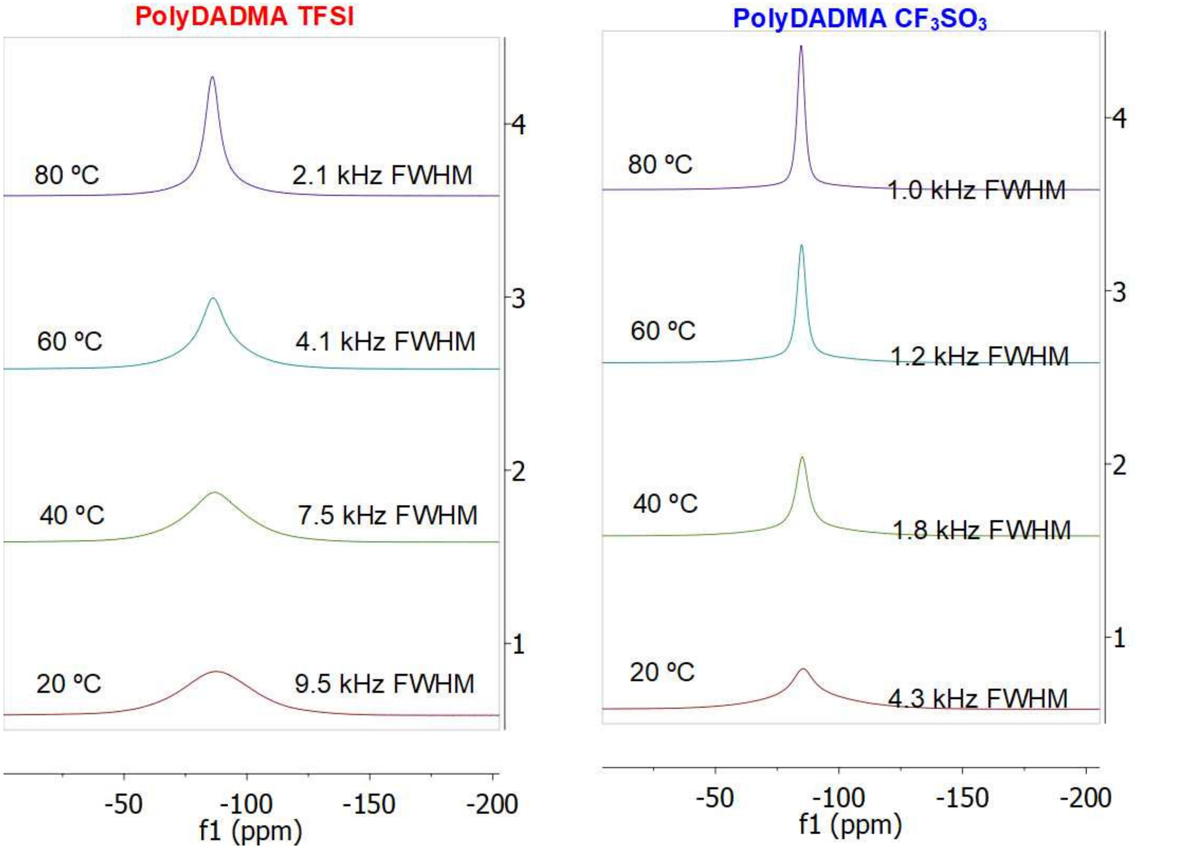

Figure S8. Static ^{19}F-NMR of PolyDADMA TFSI and PolyDADMA CF_3SO_3 at different temperatures.

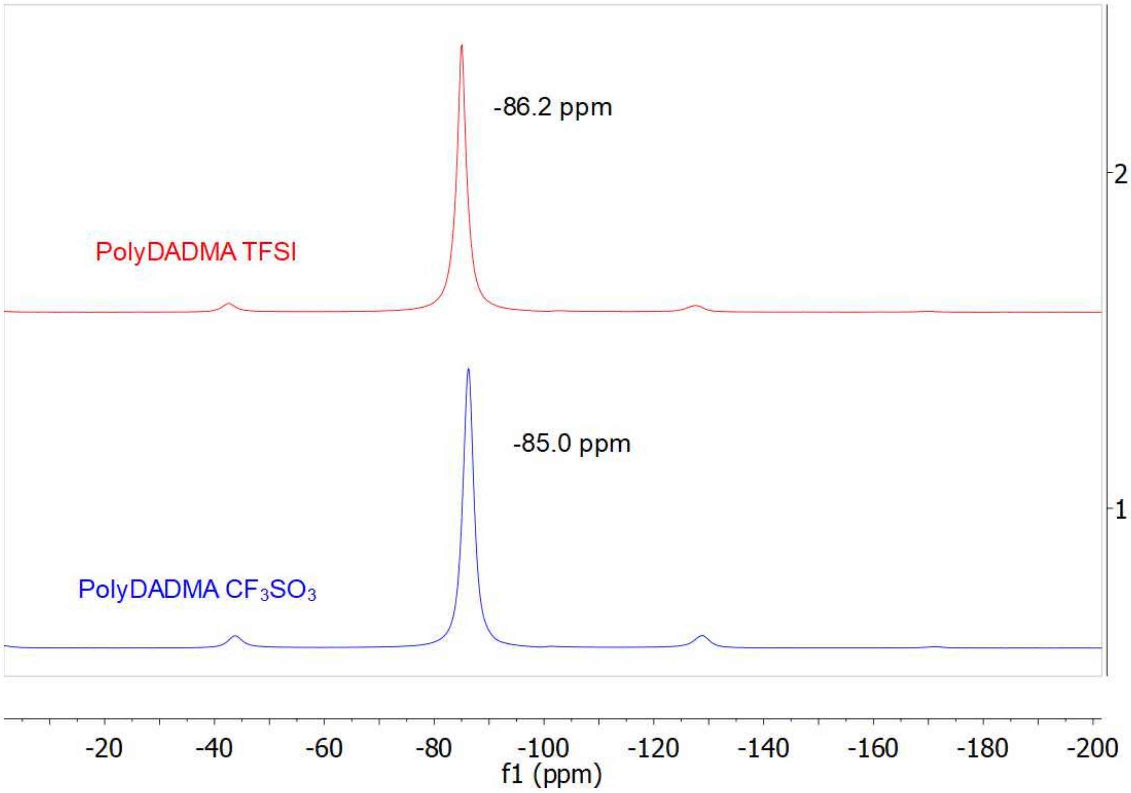

Figure S9. MAS spectra of PolyDADMA TFSI and PolyDADMA CF_3SO_3.

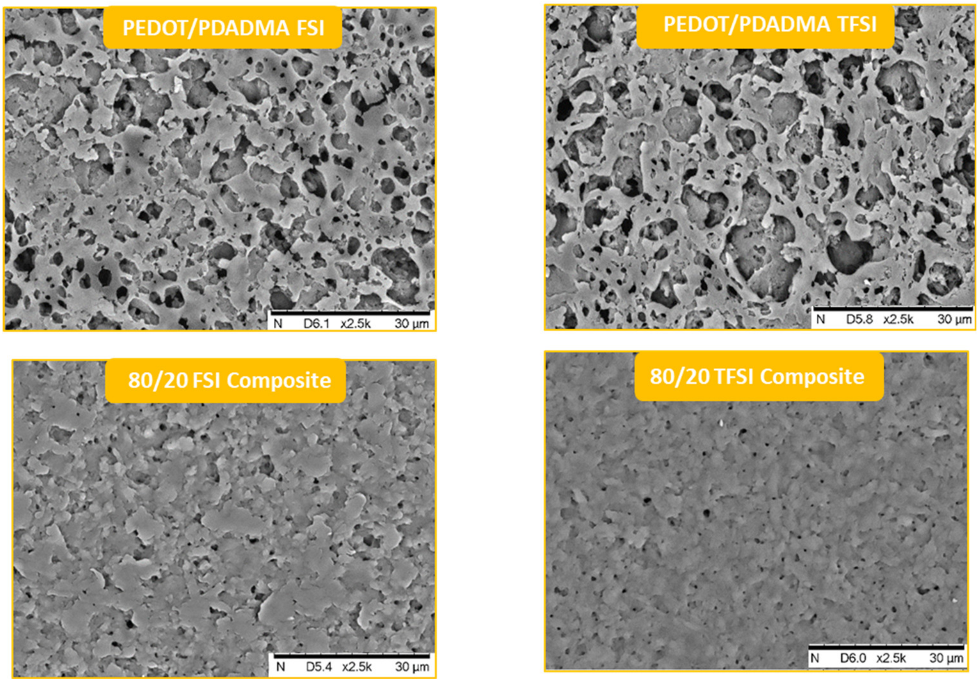

Figure S10. SEM images of PEDOT:PolyDADMA FSI, TFSI and their composites with [C_2mpyr][FSI] and [C_2mpyr][TFSI] respectively.

References

1. Mcdonald, M.B.; Hammond, P.T. Efficient Transport Networks in a Dual Electron/Lithium-Conducting Polymeric Composite for Electrochemical Applications. *ACS Appl. Mater. Interfaces* **2018**, *10*, 15681–15690.

2. Zhou, Y.; Wang, X.; Zhu, H.; Armand, M.; Forsyth, M.; Greene, G.W.; Pringle, M.; Howlett, P.C. N-ethyl-N-methylpyrrolidinium bis(fluorosulfonyl)imide-electrospun polyvinylidene fluoride composite electrolytes: characterization and lithium cell studies. *Phys. Chem. Chem. Phys.* **2017**, *19*, 2225–2234.
3. Forsyth, M.; Chimdi, T.; Seeber, A.; Gunzelmann, D.; Howlett, P. Structure and dynamics in an organic ionic plastic crystal, N-ethyl-N-methyl pyrrolidinium bis(trifluoromethanesulfonyl) amide, mixed with a sodium salt. *J. Mater. Chem. A* **2014**, *2*, 3993–4003.
4. Golding, J.; Forsyth, S.; Macfarlane, D.R.; Forsyth, M.; Deacon, G.B. Methanesulfonate and p-toluenesulfonate salts of the N-methyl-N-alkylpyrrolidinium and quaternary ammonium cations: Novel low cost ionic liquids. *Green Chem.* **2002**, *4*, 223–229.
